# Supplementary material for: Modeling glioblastoma heterogeneity as a dynamic network of cell states
Source: Mol Syst Biol. 2021 Sep 16;17(9):e10105. doi: 10.15252/msb.202010105 (PMC8444284; doi:10.15252/msb.202010105)
Supplement: Supplementary file 5 — Source Data for Figure 3 [file MSB-17-e10105-s001.zip › Figure3A_sourcedata/GSEA_3065/hallmarks_state3.GseaPreranked.1623416274744/HALLMARK_UNFOLDED_PROTEIN_RESPONSE.html]

Details for gene set HALLMARK\_UNFOLDED\_PROTEIN\_RESPONSE[GSEA]

|  || Dataset | state3 |
| Phenotype | NoPhenotypeAvailable |
| Upregulated in class | na\_pos |
| GeneSet | HALLMARK\_UNFOLDED\_PROTEIN\_RESPONSE |
| Enrichment Score (ES) | 0.34201688 |
| Normalized Enrichment Score (NES) | 1.3210093 |
| Nominal p-value | 0.044247787 |
| FDR q-value | 0.15893838 |
| FWER p-Value | 0.641 |
Table: GSEA Results Summary

  

Fig 1: Enrichment plot: HALLMARK\_UNFOLDED\_PROTEIN\_RESPONSE      
 Profile of the Running ES Score & Positions of GeneSet Members on the Rank Ordered List

  

| PROBE | GENE SYMBOL | GENE\_TITLE | RANK IN GENE LIST | RANK METRIC SCORE | RUNNING ES | CORE ENRICHMENT || 1 | HSP90B1 |  |  | 7 | 0.563 | 0.0452 | Yes |
| 2 | PDIA6 |  |  | 8 | 0.549 | 0.0901 | Yes |
| 3 | CALR |  |  | 30 | 0.473 | 0.1263 | Yes |
| 4 | HSPA5 |  |  | 43 | 0.447 | 0.1614 | Yes |
| 5 | SPCS3 |  |  | 76 | 0.370 | 0.1880 | Yes |
| 6 | FUS |  |  | 87 | 0.358 | 0.2161 | Yes |
| 7 | DNAJC3 |  |  | 182 | 0.294 | 0.2293 | Yes |
| 8 | FKBP14 |  |  | 185 | 0.292 | 0.2529 | Yes |
| 9 | SPCS1 |  |  | 194 | 0.288 | 0.2756 | Yes |
| 10 | SEC11A |  |  | 205 | 0.281 | 0.2973 | Yes |
| 11 | SRPRB |  |  | 257 | 0.260 | 0.3127 | Yes |
| 12 | ATF6 |  |  | 309 | 0.240 | 0.3265 | Yes |
| 13 | EIF4G1 |  |  | 370 | 0.226 | 0.3380 | Yes |
| 14 | MTREX |  |  | 734 | 0.166 | 0.3096 | Yes |
| 15 | SRPRA |  |  | 799 | 0.157 | 0.3151 | Yes |
| 16 | NOP56 |  |  | 829 | 0.155 | 0.3244 | Yes |
| 17 | SSR1 |  |  | 851 | 0.152 | 0.3344 | Yes |
| 18 | HYOU1 |  |  | 906 | 0.148 | 0.3403 | Yes |
| 19 | XBP1 |  |  | 1036 | 0.135 | 0.3364 | Yes |
| 20 | CNOT4 |  |  | 1082 | 0.132 | 0.3420 | Yes |
| 21 | EXOSC10 |  |  | 1355 | 0.111 | 0.3197 | No |
| 22 | SLC7A5 |  |  | 1394 | 0.109 | 0.3242 | No |
| 23 | SLC30A5 |  |  | 1496 | 0.103 | 0.3209 | No |
| 24 | KDELR3 |  |  | 1678 | 0.093 | 0.3076 | No |
| 25 | TTC37 |  |  | 1721 | 0.090 | 0.3102 | No |
| 26 | VEGFA |  |  | 1723 | 0.090 | 0.3174 | No |
| 27 | EIF4A1 |  |  | 1767 | 0.088 | 0.3196 | No |
| 28 | WFS1 |  |  | 1891 | 0.081 | 0.3121 | No |
| 29 | SLC1A4 |  |  | 2003 | 0.076 | 0.3054 | No |
| 30 | DCTN1 |  |  | 2005 | 0.076 | 0.3115 | No |
| 31 | ARFGAP1 |  |  | 2059 | 0.074 | 0.3114 | No |
| 32 | DNAJB9 |  |  | 2115 | 0.071 | 0.3109 | No |
| 33 | HERPUD1 |  |  | 2299 | 0.064 | 0.2950 | No |
| 34 | HSPA9 |  |  | 2404 | 0.060 | 0.2879 | No |
| 35 | NFYB |  |  | 2471 | 0.058 | 0.2851 | No |
| 36 | NPM1 |  |  | 2533 | 0.056 | 0.2826 | No |
| 37 | EXOSC9 |  |  | 2584 | 0.054 | 0.2812 | No |
| 38 | EXOC2 |  |  | 2593 | 0.054 | 0.2847 | No |
| 39 | EDEM1 |  |  | 2724 | 0.049 | 0.2737 | No |
| 40 | ERO1A |  |  | 3035 | 0.040 | 0.2412 | No |
| 41 | EIF2S1 |  |  | 3099 | 0.038 | 0.2370 | No |
| 42 | ALDH18A1 |  |  | 3109 | 0.038 | 0.2391 | No |
| 43 | NOP14 |  |  | 3158 | 0.037 | 0.2366 | No |
| 44 | EIF4A3 |  |  | 3479 | 0.029 | 0.2019 | No |
| 45 | STC2 |  |  | 3529 | 0.027 | 0.1985 | No |
| 46 | EXOSC1 |  |  | 3646 | 0.024 | 0.1871 | No |
| 47 | TUBB2A |  |  | 4022 | 0.015 | 0.1450 | No |
| 48 | KIF5B |  |  | 4156 | 0.011 | 0.1305 | No |
| 49 | EXOSC2 |  |  | 4160 | 0.011 | 0.1310 | No |
| 50 | ASNS |  |  | 4228 | 0.009 | 0.1240 | No |
| 51 | PARN |  |  | 4230 | 0.009 | 0.1246 | No |
| 52 | YIF1A |  |  | 4481 | 0.002 | 0.0958 | No |
| 53 | SHC1 |  |  | 4506 | 0.001 | 0.0932 | No |
| 54 | SDAD1 |  |  | 4518 | 0.001 | 0.0920 | No |
| 55 | DCP1A |  |  | 4544 | 0.000 | 0.0891 | No |
| 56 | GEMIN4 |  |  | 4648 | -0.002 | 0.0774 | No |
| 57 | DDX10 |  |  | 4704 | -0.004 | 0.0713 | No |
| 58 | KHSRP |  |  | 4723 | -0.004 | 0.0696 | No |
| 59 | DCP2 |  |  | 4950 | -0.010 | 0.0442 | No |
| 60 | DDIT4 |  |  | 5025 | -0.012 | 0.0366 | No |
| 61 | SEC31A |  |  | 5142 | -0.014 | 0.0244 | No |
| 62 | EEF2 |  |  | 5276 | -0.018 | 0.0105 | No |
| 63 | TATDN2 |  |  | 5368 | -0.020 | 0.0016 | No |
| 64 | XPOT |  |  | 5548 | -0.026 | -0.0170 | No |
| 65 | RRP9 |  |  | 5569 | -0.026 | -0.0171 | No |
| 66 | CNOT2 |  |  | 5714 | -0.030 | -0.0313 | No |
| 67 | EIF4E |  |  | 5835 | -0.034 | -0.0424 | No |
| 68 | ATF3 |  |  | 6159 | -0.045 | -0.0760 | No |
| 69 | ZBTB17 |  |  | 6167 | -0.045 | -0.0731 | No |
| 70 | NOLC1 |  |  | 6241 | -0.048 | -0.0777 | No |
| 71 | DKC1 |  |  | 6315 | -0.050 | -0.0820 | No |
| 72 | CXXC1 |  |  | 6351 | -0.051 | -0.0819 | No |
| 73 | PREB |  |  | 6379 | -0.052 | -0.0808 | No |
| 74 | BAG3 |  |  | 6417 | -0.053 | -0.0807 | No |
| 75 | EIF4A2 |  |  | 6481 | -0.056 | -0.0834 | No |
| 76 | WIPI1 |  |  | 6485 | -0.056 | -0.0792 | No |
| 77 | EXOSC5 |  |  | 6616 | -0.062 | -0.0892 | No |
| 78 | MTHFD2 |  |  | 6745 | -0.068 | -0.0984 | No |
| 79 | ATF4 |  |  | 6789 | -0.070 | -0.0976 | No |
| 80 | CEBPG |  |  | 6829 | -0.072 | -0.0962 | No |
| 81 | ATP6V0D1 |  |  | 6854 | -0.073 | -0.0930 | No |
| 82 | CEBPB |  |  | 6863 | -0.074 | -0.0879 | No |
| 83 | CNOT6 |  |  | 7235 | -0.096 | -0.1229 | No |
| 84 | GOSR2 |  |  | 7420 | -0.110 | -0.1352 | No |
| 85 | POP4 |  |  | 7450 | -0.113 | -0.1293 | No |
| 86 | CKS1B |  |  | 7569 | -0.124 | -0.1328 | No |
| 87 | PSAT1 |  |  | 7634 | -0.131 | -0.1295 | No |
| 88 | PAIP1 |  |  | 7795 | -0.151 | -0.1357 | No |
| 89 | EXOSC4 |  |  | 8026 | -0.189 | -0.1468 | No |
| 90 | LSM1 |  |  | 8029 | -0.189 | -0.1316 | No |
| 91 | IMP3 |  |  | 8041 | -0.192 | -0.1172 | No |
| 92 | YWHAZ |  |  | 8166 | -0.220 | -0.1136 | No |
| 93 | SERP1 |  |  | 8229 | -0.235 | -0.1016 | No |
| 94 | EIF4EBP1 |  |  | 8316 | -0.262 | -0.0901 | No |
| 95 | BANF1 |  |  | 8514 | -0.345 | -0.0847 | No |
| 96 | LSM4 |  |  | 8611 | -0.413 | -0.0620 | No |
| 97 | NHP2 |  |  | 8632 | -0.436 | -0.0287 | No |
| 98 | RPS14 |  |  | 8700 | -0.518 | 0.0059 | No |
Table: GSEA details [plain text format]

  

Fig 2: HALLMARK\_UNFOLDED\_PROTEIN\_RESPONSE: Random ES distribution      
 Gene set null distribution of ES for **HALLMARK\_UNFOLDED\_PROTEIN\_RESPONSE**
